# Strabismus

**Published:** 1870-08

**Authors:** H. W. Boyd

**Affiliations:** Prof. Anatomy, Chicago Medical College


CHICAGO MEDICAL EXAMINER.

N. S. DAVIS, M.D., Editor.

VOL. XI. AUGUST, 1870. NO. 8.

### Original Contributions.

### ARTICLE XVIII.

### STRABISMUS.

By H. W. BOYD, M.D., Prof. Anatomy, Chicago Medical College.

Strabismus consists in a lack of parallelism in the axis of vision, so that it is impossible to direct both eyes to the same point simultaneously. This has always been considered mere deformity, owing to the contraction of a tendon, but of late years, the pathology of the affection has been carefully studied, and shown by Donders to be something more. The contraction of the tendon is not the primary, but simply a secondary cause, and at first merely a symptom of the original or primary difficulty. Strabismus affords a wide field for study, and is one of the most abstract and complicated subjects connected with ophthalmic science. The true pathology of the affection was never understood until the subject of the anomalies of refraction attracted the attention of Donders and his earnest co-laborers. Within a few years, writers have ascribed as the causes of strabismus, "imitation of other squinting persons," to convulsions, intestinal worms, etc., the category of causes being unlimited, including every disease, accident, and incident of any kind, which might have happened about the time the affection first appeared, never explaining how any of the causes operated in producing the affection.

There are four forms of strabismus described. Strabismus convergens, in which the deviation is inwards. Strabismus divergens, in which the deviation is outwards, and also two other forms—one in which the eye deviates upwards, and in the other downwards, (S. subvergens, and S. deosubvergens): the two latter forms are so rare that they are seldom seen. Convergent strabismus is an abnormal deviation of one or both eyes inwards. This is by far the most common form of squint, and it is easy to see that the axis of vision of the two eyes not being parallel, but one eye can be used at a time in the act of seeing, and in the early stages, the patient either suffers double vision, or, he converges the eyes so much that he actually becomes a one-eyed person, so far as actual vision is concerned: the diplopia, however, lasts but a few months, for by its continual disturbance of vision, the eye is thrown permanently aside, and the prolonged disuse of the organ entails such a degree of amblyopia that it becomes useless, unless the affection is double and equal in each eye; in this case he uses one eye as much as the other alternately, first one and then the other, in which case vision is preserved equally in both eyes.

Strabismus exists in various degrees, and it is frequently desirable to measure the extent of the deviation; this may be accomplished in a variety of ways. A simple method is to have the patient look at some fixed point, and, of course, his sound eye being fixed upon one point, the affected eye will be at its greatest convergence or divergence, as the case may be; then with a pen make a mark on the lower lid, just below the centre of the pupil, then let the patient close his sound eye with his fingers, and fix the object with the affected eye, and when the eye is fixed make a second mark just below the center of the pupil, and the distance between these two points marked on the lower lid, measured in lines, will express the degree of the strabismus. This is a simple method of measuring the degree of the deviation, and is very convenient when the surgeon does not possess an instrument called the strabismometer. The one invented by Mr. Lawrence is a very good one, and is the one now in general use ; it consists of a plate of ivory made to fit closely to the lower lid, the outer side of it is graduated in lines and half lines ; this plate is fitted into a handle, and by placing it to the lower lid just below the eye, it is easy to see that the position of the cornea can be ascertained exactly, and the extent of the deviation shown, either in the convergent or divergent forms ; it should be tested for both near and distant objects, as it may be considerably greater during a strong effort of accommodation, as reading very fine print, than when the patient is looking at a distant object. In some cases of stabismus, the eyes do not seem to be in the same plane, one eye seems to be higher than the other; this may be owing to the upper fibres of the internal rectus being contracted more than the lower or middle fibres of the same muscle, or it may be owing to the fact that the superior rectus is also implicated in the affection ; this point must be accurately determined, by examining into the associated action of the two eyes. The sound eye must be covered with the hand, and the affected eye be made to fix upon some object; now, if the internal rectus be the only one affected, the associated action of the sound eye will be exactly lateral, without any deviation in height; while if the superior rectus is also implicated the sound eye will not only make a lateral movement, but also a downward movement, corresponding to the downward movement of the other eye. In the first instance, the strabismus may be cured by tenotomy of the rectus internus, and especially dividing pretty freely towards its upper border; but in the last, only by a tenotomy of the whole of the superior rectus.

The mobility of eyes affected with strabismus is not materially affected, except in some very old aggravated cases. In alternating strabismus, so called because the squint alternates, first in one eye, and then in the other, the visual power is equal in each eye, and the motive power in such cases is never decreased. The arc of mobility is the same in both eyes, only it is shifted inwards, or outwards on the affected side, according as the case is convergent or divergent ; but this increase of mobility on the side of the shortened muscle, is counterbalanced by a corresponding loss in the movements outwards, in convergent, and the movements inwards, in divergent strabismus. If we hold a pencil or any object in front of the eyes of the patient, and move it from side to side in front of his face, it will be seen that both eyes follow the object in its movement, the affected eye always holding the same associated relation to the sound one, always having the same angle of deviation, except when directed to objects situated at great distances, and for this reason this form of squint is called by Von Græfe concomitant. In strabismus resulting from paralysis of the externus or internus, of course the motor power of the eye is lost, and all associated movements are destroyed in the direction of the paralyzed muscle, and according to Von Græfe, it is even difficult for the sound eye to make certain associate movements, and consequently the fixation of objects in certain positions can be kept up with great difficulty, or is not undertaken at all; and then the patient tries to assist himself by turning his face to the side towards which the movement of the one or the other eye is rendered difficult, and in this way lessons the demand on the expenditure of power of the affected. muscle; and it is also observed, in these cases where the head is turned to one side, that the degree of the strabismus is apparantly somewhat lessened. The patient is also usually aware of advantages arising from certain positions of the head, in apparently lessening the deformity, and by habitual practice he gradually accustoms himself to a very peculiar manner of holding his head, which in a few years becomes a permanent position of the head. The muscles of the neck are in an abnormal relationship to these of the eye, and will frequently continue so, even after successful operations for the cure of the strabismus. It is a question of considerable interest as to what extent binocular vision exists in strabismus, or rather the possibility of obtaining it after an operation; its presence is proved by the existence of binocular diplopia. Cases are occasionally operated upon where the diplopia annoys the patient for weeks, or until binocular single vision is produced by means of glasses; such causes are rare. It is quite common where you get binocular vision, for the diplopia to annoy the patient for a few days, but it soon passes away, and leaves the patient with binocular single vision. According to Stellwag, binocular vision is but rarely, if ever, at all obtained; while on the other hand, Von Græfe claims that at least fifty per cent of the cases have binocular single vision. Thus it would seem that considerable discrepancy exists between high authorities on this point. Stellwag claims that the data from which Von Græfe forms his opinion are not reliable; while his own data are derived from the falling experiment of Hering. Græfe supports his views by experiments with the stereoscope. It is certain that both of these distinguished gentlemen are too radical on this point. Græfe says that even before the operation is performed, with the organ in its abnormal position, that the perceptive power is assisted quite considerably by the squinting eye, not only in the lateral qualitative perceptions, but by markedly increasing the intensity of the fixating eye. Cases are often seen where binocular vision results from properly performed operations, and also many cases where it is impossible to obtain it, even when the eyes were apparently perfectly parallel. It is believed that nearly half or at least one-third of the cases operated upon have binocular vision. It depends, according to some, upon the age at which the operation is performed, or rather at the age of the squint. An early operation stands the best chance for binocular vision, while those cases operated upon late in life, rarely succeed in obtaining it. The object aimed at in an operation should be perfect parallelism and binocular vision; and although there may be considerable amblyopia from the prolonged disuse of the eye, and you may not get immédiate binocular vision, it is possible to obtain it by systematic use or exercise of the eye, by binding up the sound eye and educating the affected one at certain hours in the day. Good results from this course are seen in children and young persons where there was considerable amblyopia. In cases where binocular vision cannot be obtained, an operation can only be resorted to as a means of improving the appearance.

According to Stellwag, periodical strabismus is the only kind in which binocular vision ever exists, and consequently the only kind in which tenotomy can be regarded as the true means of cure. But there are many well-authenticated cases, in which binocular vision, though lost for years, was regained. This is a point upon which too little attention has been bestowed. We are all too fond of operating, simply to get "them straight," and allowing our patient to leave us—perhaps to see him no more, without even a single trial with prisms or glasses, not even thinking of whether the patient sees with one or both eyes. Our object should be to use all our skill, both surgical and therapeutical, to obtain for the affected eye, its share in the act of vision, and not be satisfied with simply rendering the "eyes straight,"—the truth is, too much attention has been paid to it simply as a deformity, and altogether too little to the cause, or even the effect, of the deviation in producing the amblyopia.

It appears somewhat difficult sometimes for the surgeon to diagnosticate which eye is most affected; generally a little thought on the subject will enable him to decide the point. The difference in the visual power of the two eyes should be first examined, and frequently it will be found that the squinting eye is nearly blind. Next examine the abductive and adductive power of the two eyes, according as the case may be convergent or divergent. There are many cases in which the cornea can be almost hid in the inner angle of the eye. Next examine the palpebral fissure; it is sometimes smaller in the affected eye, than in the sound eye, on account of the person closing his eye —especially when he is in a bright light—unconsciously, and to obviate the confused vision, arising from double images.

The pathology of strabismus has recently received a great deal of attention, and has been thoroughly revolutionized and changed. None of the old writers on the eye seem to have comprehended the subject in its true scientific bearings. It is now shown, that in its early stages, it is merely a symptom, having intimate relations with the functions of the eye, as an organ of vision, and, in time, that it may cause the total extinction of the visual power. The power of the organ to see gradually declines, because the visual axes cannot be brought to bear upon the same point simultaneously. This causes confusion of vision; images being formed upon parts of the retina which do not correspond; and to prevent this confusion, arising from double vision, binocular vision is dispensed with. The image formed upon the retina of the affected eye, being received at some point less favorable than the macula lutea (the visual axis corresponding to lines drawn from the macula lutea, in each eye, through the optic center, towards the object looked at), is disregarded in the sensorium; and so it continues until vision is extinct, from a lack of exercise. Nearly all the cases of convergent strabismus are caused by the anomaly of refraction, known as hypermetropia—a condition of the eye in which the antero-posterior diameter is shortened; the principal focus for the rays of light coming from distant objects is not made on the retina, but behind that membrane. This is the true opposite of myopia. For many years, presbyopia was supposed to be the true opposite of myopia; but it has been shown by Donders, that presbyopia is occasioned by an increase of hardness in the crystalline lens, so that its form can no longer be changed, and its convexity be readily increased by the action of the ciliary muscle, which causes a recession of the new point of sight, while the refractive power for distant objects remains undiminished.

Prof. Donders was the first to make known the fact, that this condition of the eye gives rise to convergent strabismus. A person affected with hypermetropia depends upon his accommodative powers for distinct vision, either near or distant. Accommodation consists in the action of the ciliary muscle causing a change in the curvature of the crystalline lens, thereby changing the condition of refraction. Now, keeping in mind the fact, that in hypermetropia, the eyeball is shortened, and, in looking at near objects, the patient tries to strengthen his powers of accommodation, by increased convergence of the axis of vision, and the increased exercise which the internal rectus gets during this forced convergence, being almost constant, soon increases the strength of the muscle, and produces spasm; and the strabismus, at first occasional, finally becomes permanent; and, in accordance with this, we find that convergent strabismus oftenest begins to show itself in children, at about three or four years of age, when they first begin to use their eyes much upon near objects. And it has also been observed, that in those portions of the country where but little attention is given to the education of the young, and where there is no necessity for using the eyes much upon near objects, that convergent strabismus is much less frequent than in the cities and towns, where considerable attention is given to the education of the young. Hypermetropia is frequently hereditary, and it is a common circumstance to find several members of the same family affected with it; and this will also account for the popular belief that strabismus is caused by imitation, or by the child looking at some older member of the family who has strabismus. Nine members of one family were all affected with strabismus; and according to the popular belief of the family and friends, each one acquired it from imitating or looking at the next older one. Not every person who is hypermetropic has strabismus. According to Donders, not even persons with the same degree of hypermetropia, and of the same occupation, in fact, only a small percentage of hypermetropes squint. It requires only the milder forms of hypermetropia to produce strabismus, and it is seldom, if ever, found in the higher degrees. This is for the reason that the power of accommodation is insufficient to produce a perfect image upon the retina, and the patient accustoms himself to gain correct ideas from imperfect representation, rather than improve them by a maximum of effort (Donders). Convergent strabismus is also, though rarely, caused by a myopic form of the eyeball. Myopia is that form of the eye in which its antero-posterior diameter is elongated, and the retina is situated behind the main focus; parallel rays, coming from distant objects, converge in front of the retina, in the vitreous humor. Where convergent strabismus occurs, it is only in the milder forms of myopia, and when the eyes are continually employed with small objects; and on account of the error in refraction, great convergence is required; and, thus, by continued exercise, the internal recti become hypertrophied, and overbalance the external, by increased strength. Now, in the stronger degrees of myopia, the necessary convergence is generally very difficult, and the patient fixes near objects with but one eye; and this is the reason why convergent strabismus is confined to the milder forms of myopia.

Convergent strabismus also results from paralysis of the rectus externus. When this is the case, the external rectus undergoes structural changes, and alters the original conditions of association, and, consequently, although the paralysis may be cured, the internal rectus remains the same contracted muscle. Cases where the eye turns upwards or downwards are probably always caused by this condition. Opacities of the cornea sometimes produces a convergent strabismus. It is commonly said, that the eye seeks this position, to bring some transparent portion of the cornea into the axis of vision, so that binocular vision could be preserved. But this is evidently a mistaken view; for if such cases are carefully examined, it will be found that no binocular vision exists, and that the affected eye is of no use to the patient, in any position whatever, and that it has probably never been used in the act of vision, since the opacity made its appearance. The opacity probably interfered with the correct vision, and the eye was converged, simply to prevent confusion, or diplopia, and remained so. It is also a common belief that strabismus is sometimes caused by the retina becoming insensible, in certain spots, and that the eye converges to bring some more sensitive spot into the axis of vision. This, also, is a mistaken view; as, in such a case, the point of convergence for rays of light would not correspond on the retina of the two eyes. The vision would be confused, and the affected eye would be thrown aside, only one eye being used in vision.

Divergent strabismus is that form of the affection, in which the deviation is outwards. It is much more rare than the convergent, occurring in the proportion of about one to twenty of the latter, excepting those cases which occur as a secondary result from an operation for the convergent form. More than two-thirds of all the cases of divergent strabismus originate in a myopic form of the eyeball, which, as stated above, is the opposite of hypermetropia. It is said that this form of strabismus does not appear until later in life than is usual for the convergent. Myopia is frequently caused by a posterior staphyloma, or a relaxing of the posterior walls of the globe, thus giving to the eye an elongated shape and increasing its antero-posterior diameter, interfering materially with the actions of the muscles. It is plain that they must act at a great disadvantage on a globe, of nearly the same shape as the orbital cavity itself. In examining near objects, a greater convergence is required, in proportion as the object is brought nearer to the eye; and, in myopia, the eyes are kept in a constant strain, in the attempt to keep up the necessary convergence, until a tireing of the internal recti takes place, and we have a condition known as asthenopia. It is then impossible for the eye to further participate in the attempt at convergence, and, hence, but one eye is employed in the act of vision; and the other eye is allowed to deviate outwards, because he finds that the attempt to keep up the convergence only induces weariness of the internal recti and of the ciliary muscle, without actually attaining the advantages of binocular vision (*Williams*). If the myopia is progressive and increases rapidly, the internal recti are certain to become tired out, and the divergence to occur. But, on the other hand, if the myopia increases very gradually, the internal recti may gain so much power, by their exercise in convergence, that they meet the demand made upon them without difficulty. At first, the divergence shows itself only when the eye is used upon very near objects, or when the internal rectus is very much enfeebled from use. But as the enfeebled condition of the internal rectus becomes greater, the divergence becomes more decided and permanent. This is owing to the strong efforts required in convergence, calling into play an act of accommodation, on the part of the ciliary muscles, which brings the far point yet nearer, and renders the perception of objects less clear. This not being pleasant to the patient, he endeavors to avoid it by giving up binocular vision and contenting himself with a clearer image, formed by a single eye. He encourages an outward deviation unconsciously, so that he may not be annoyed by double images, which constantly trouble him, in his efforts to produce convergence, sufficient to have an image upon corresponding portions of the retina of the two eyes.

Much has of late been said in relation to the cure of this affection. In cases caused by the anomalies of refraction, great attention should be paid to the cause of the strabismus. Some of the best authorities recommend a prophylactic treatment, directed immediately to the causes producing it. When hypermetropia is a cause, the disease makes its appearance when the child first begins to use its eyes upon near objects, at about four or six years of age. Prophylactic treatment consists, *first*, in *preventing* the child from using its eyes upon near objects, and allowing it to be taught reading, writing, seeing, etc., etc., at a later period; and, *second*, in neutralizing the hypermetropia with a suitable convex lens; not allowing the tender age of the patient to prevent the use of such glasses. Strabismus may be prevented in this way, and cases of periodical strabismus entirely cured. Heretofore, it must be acknowledged too little attention has been paid to the causes of strabismus. It has been considered too much as a mere deformity, as simply a contracted muscle, and has been treated accordingly. From what is now known of the nature of strabismus, it is believed that many cases may be cured simply by lessening the work of the internal rectus, which can be accomplished by modes of amusement or study, in which the eye will receive exercise. Even if it has become permanent, it may sometimes be rectified, when not of long standing, by simply keeping away the exciting causes which originally produced the deviation; or, at least, the angle of the squint may be considerably lessened. It is not an unfrequent occurrence to meet persons who squinted badly in their younger days, but have overgrown it, and whose eyes are now quite, or nearly straight; but an examination will show most of them to be blind in the affected eye. Such cases are those who have used correcting glasses in season, or have given up the use of their eyes upon near and small objects, continued reading, writing, etc. There is no doubt that many cases, if seen in time, might be treated successfully. The old plan of black spots upon the temples, and, also, of spectacle goggles, with the inner half of the glass opaque, the design being to cause the patient to look outwards, is of no benefit whatever, and, in fact, will have a stronger tendency to aggravate the disease and make it worse than to cure it. The ordinary method of curing strabismus at present, after all other methods have failed, is to perform tenotomy, upon the tendon of the contracted muscle.

This operation was first performed in 1737, by the celebrated charlatan oculist *Taylor*, who pretended to the world that he had miraculous powers. He had announced in the *Mercure de France*, June, 1737, "Dr. Taylor, oculist to the King of Great Britain, has just arrived in Paris, residing at the London Hotel, Dauphine Street, where he will remain until July, when he goes to Spain. He asks us to publish the discovery which he has made of the rectification of squinting eyes, by means of a simple and almost painless operation, unattended with any danger of accident." It is conceded by the best authorities, that his operation consisted in the division of the lateral recti muscles. *Huerman*, of Leipsic, on the "Newest Surgical Operations," condemned the operation; and it was entirely abandoned from that period, until, in 1838, when Stromerger, of Hanover, published a paper upon the subject. He said he had not performed the operation upon the living subject, but had demonstrated its feasibility upon the cadaver. And in October, 1839, the first operation for strabismus was performed by Dieffenbach and Curiver, simultaneously; although the united voice of the profession awards Dieffenbach the honor of performing the first operation. After this, the operation, although at first enthusiastically taken hold of, fell into some disrepute, on account of the signal failure of several eminent surgeons. Velpeau failed in six or eight cases; and the French surgeons were on the point of abandoning the operation as a failure, when Phillips, an English surgeon, came to Paris, and finding this state of affairs, he performed the operation upon several cases, with the most triumphant success, in the presence of the Academy of

Medicine. Amusart, Lallemand, Pinel, Grandschamp, Lisfranc, and Velpeau were all present, and witnessed the operation, and the Academy unanimously accorded its favor.

There are many modifications of the operation; and to do it really well—to liberate the eye with certainty and in an efficient manner, so as to secure the utmost benefit capable of being conferred, at the smallest possible cost of suffering and inconvenience to the patient—requires much more dexterity, neatness, and address than is commonly believed. Every tyro in surgery thinks he can perform it with success; but the truth is, but very few succeed in getting a good result in any great number of cases, until they have attained a delicacy and excellence to be got only from operating upon a large number of cases. But very few have ever performed the operation to any extent, who have not had their "*only partial*" successes, or total failures, or who have not occasionally made the eye look much worse than the original deformity, by an unskilfully performed operation. This may, however, be partially avoided by a minute knowledge of the anatomy and pathology, and also of each of the steps in the operation.

The question as to what age is most suitable for this operation, is one of some importance. The highest authorities disagree in regard to it. Some prefer to operate at from four to six years of age, because at this period the child is first using its eyes upon near objects, and that one or both eyes are apt to suffer from disease, by being thrown to one side, thus producing amblyopia; while it is claimed by others that the eyes are apt to diverge when operated upon at so young an age, and that it is better to risk the amblyopia, and wait until the strabismus has become more permanently established, and in the meantime make use of all the prophylactic measures possible, and thus try to cure the case without tenotomy. If an operation is performed at six years of age, the cause still exists, and the patient would suffer from diplopia. Stellwag recommends the prophylactic treatment to be continued for years, and that a patient may grow out of a confirmed strabismus. The operation should be postponed until at least nine or ten years of age. Nothing can be gained by an early operation.

Tenotomy may be performed in any kind of weather, without regard to season, or any material preparation on the part of the patient. An anæsthetic may, or may not be given, according to circumstance. The operation can be performed with much greater certainty of success, and better satisfaction to both patient and surgeon, without it. The patient can co-operate with the surgeon, and assist him in obtaining a perfect result; whereas, under the influence of chloroform or ether, the patient does not co-operate with the surgeon, and it is impossible for the surgeon to tell what effect he has produced until the patient wakes up, and he finds frequently that he has either done too much or not quite enough, and he must administer more of the anæsthetic, or else wait and make another operation.

In regard to the position of the patient, the best is for him to sit up, perfectly erect, with the body inclined a little towards the operator, and an assistant standing behind to steady his head, and to hold it in the proper position. The patient's hands must be close down by his sides. Holding tightly to the chair in this position, with his mind made up to endure the pain with fortitude, there is generally but little trouble in getting an excellent result.

The instruments required for this operation are as follows: Pair of mouse-toothed forceps, a spring-wire speculum for holding open the eyelids, a pair of small sharp scissors (either curved or straight), and a blunt hook. These four instruments are all that are necessary. The speculum should be introduced, and the lids separated. A small fold of the conjunctiva should be caught up with the forceps near the lower margin of the rectus muscle, and a very small incision made in it with the scissors, and the scissors should then be pushed in and be made to separate the conjunctiva from the capsule of Tenon which is adherent to the muscle or tendon, as well as to the conjunctiva. After the separation is completed, the blunt hook should be introduced, and the tendon pulled down, and with the scissors it should be divided up close to the sclerotic. The speculum should then be taken from the eye, and the organ bathed with cold water, and allowed to rest a while; after resting it should be examined, and if it is not straight then the patient resumes his position, and the speculum is introduced. The blunt hook is then swept under the conjunctiva, and other muscular fibres, or the parts which continue to hold the eye in its abnormal position, are dragged down to the little opening in the conjunctiva, where they are divided with the scissors, as before, and so on, until the eyes are in a suitable position. Sometimes it is necessary to have the patient sit four or five times, and allow this process to be repeated. Sometimes persons are found where it is impossible to bring the eyes perfectly parallel at one operation. In such cases it is necessary to perform two operations, with several weeks or months intervening between them. After the operation, no treatment is generally necessary, as the parts will recover their natural whiteness and appearance in about fourteen days. It is important to make as small an incision in the conjunctiva as possible; too free division of the conjunctiva would have the effect of allowing the eye to protrude, and appear larger than the sound eye. This abnormal prominence can not be remedied. Too free division of the conjunctiva may also allow the eye to diverge, this giving rise to the opposite deformity. As a rule, it may be depended upon, that the smaller the incision through the conjunctiva the better, especially in the slight forms of strabismus. When the tendon has been divided close up to the sclerotic, it slips backwards, and unites again to the sclerotic a few lines back of its original attachment, (according to the degree of deviation).

It is the practice of some surgeons to use leather spectacles, or goggles, made expressly for strabismus cases, just after the operation, so as to insure success by keeping the eyes straight, or, if they are not perfectly straight, to bring them so. The spectacles are made of dark or opaque glass, with a hole in the centre to admit the light, or if, from an accident in dividing too much tissue in the operation, the eye should diverge a little, the aperture is made more internal, to cause the eye to turn inwards, and thus bring the eyes straight. This is totally useless and unscientific. I cannot see how any one, understanding the nature of strabismus, could resort to such a practice. With such spectacles, the patient could use only one eye, just as he did before the operation, and the aperture in the spectacle would remain unnoticed.

A frequent result of the operation is sinking in of the caruncle, so as to present an ugly little pit or depression at the inner canthus. This is caused by the tendon and conjunctiva both being attached to the capsule of Tenon, which is connected anteriorly with the conjunctiva and posteriorly with the tendon, forming a sheath for it. When the tendon is divided, the muscle contracts and draws the capsule with it; and if the conjunctiva is allowed to remain in connection with it, it pulls it back, and the caruncle of course would go with the conjunctiva. The only way in which to avoid this, is to make pretty free division between the conjunctiva and capsule before the tendon is divided.

There are many modifications of the operation for strabismus, but the one described is that usually performed by a number of the best surgeons, and is the one best calculated to give good results in the largest number of cases. The operations are all described in the standard works on the eye. The literature of the operations for strabismus is perfectly enormous.

Now, after the operation has been performed, we generally leave the original cause in existence; and the question is, what shall be done to prevent a return of the affection. Many cases of convergent strabismus have their vision very greatly improved immediately after the operation. No doubt that in these cases, the hypermetropic shape of the eyeball is relieved by the tenotomy freeing the eye from the tension of the internal rectus. I have had many patients who have experienced very great improvement in vision immediately after the operation is performed. The practice of using glasses after the operation, has never received the attention it deserves from the profession. There are certainly a large number of cases which would derive great benefit from the use of glasses, and especially cases of divergent strabismus dependent upon progressive myopia. In such cases there is an imperative demand for concave glasses, because they not only very greatly improve the patient's vision, but they also have a tendency to arrest the course of the disease, which consists in a thinned or relaxed condition of the posterior walls of the eye, and a consequent protrusion backwards, constituting posterior staphyloma. It is also best to use convex glasses in cases of convergent strabismus, for by this means we have the best chance to secure the neutralization of the anomaly of refraction, and also to secure binocular vision.

